# Novel pre-treatment of zeolite materials for the removal of sodium ions: potential materials for coal seam gas co-produced wastewater

**DOI:** 10.1186/s40064-016-2174-9

**Published:** 2016-05-10

**Authors:** Oscar Santiago, Kerry Walsh, Ben Kele, Edward Gardner, James Chapman

**Affiliations:** School of Medical and Applied Sciences, CQUniversity, Rockhampton, QLD 4701 Australia; Arris Pty Ltd, Rockhampton, QLD Australia

**Keywords:** Coal seam gas (CSG), Ion exchange, CSG water management, Water treatment, Saline water, Zeolite

## Abstract

Coal seam gas (CSG) is the extraction of methane gas that is desorbed from the coal seam and brought to the surface using a dewatering and depressurisation process within the saturated coalbed. The extracted water is often referred to as co-produced CSG water. In this study, co-produced water from the coal seam of the Bowen Basin (QLD, Australia) was characterised by high concentration levels of Na^+^ (1156 mg/L), low concentrations of Ca^2+^ (28.3 mg/L) and Mg^2+^ (5.6 mg/L), high levels of salinity, which are expected to cause various environmental problems if released to land or waters. The potential treatment of co-produced water using locally sourced natural ion exchange (zeolite) material was assessed. The zeolite material was characterized for elemental composition and crystal structure. Natural, untreated zeolite demonstrated a capacity to adsorb Na^+^ ions of 16.16 mEq/100 g, while a treated zeolite using NH_4_^+^ using a 1.0 M ammonium acetate (NH_4_C_2_H_3_O_2_) solution demonstrated an improved 136 % Na^+^ capacity value of 38.28 mEq/100 g after 720 min of adsorption time. The theoretical exchange capacity of the natural zeolite was found to be 154 mEq/100 g. Reaction kinetics and diffusion models were used to determine the kinetic and diffusion parameters. Treated zeolite using a NH_4_^+^ pre-treatment represents an effective treatment to reduce Na^+^ concentration in coal seam gas co-produced waters, supported by the measured and modelled kinetic rates and capacity.

## Background

Coal seam gas (CSG), also known as coal-bed methane (CBM) is gas adsorbed onto underground coal seams and is composed mainly of methane, originating from biogenic, thermogenic and metamorphic sources (Nghiem et al. 2011). In Australia, large CSG deposits are found in Bowen and Surat coal basins of QLD (Baker and Slater 2008). The production of CSG is achieved by allowing methane gas to desorb from the coal seam and flow to the surface, which results in large volumes of co-produced water from the saturated coal seam. In addition, CSG recovery results in virtually no negative impact on future extractive mining of the coal deposits (Baker and Slater 2008). The large volumes of co-produced water in the CSG operation presents a significant challenge to manage, especially for future use. It is estimated that over the next 25 years the CSG industry will produce an average volume of 25 gigalitres (GL) of water per year in QLD’s gas fields (Hamawand et al. 2013).

CSG co-produced water has a geochemical signature characterised often by high levels of salinity, high concentrations of sodium ions and dissolved trace metals that cause various environmental problems if released to land or waters without treatment or incorrect management (Jackson and Reddy 2007; Jones et al. 2014; Regan et al. 2013). On the other hand, CSG co-produced water has the potential to be used for these beneficial usages including: irrigation, feedlots watering, aquaculture and agricultural activities (Wang et al. 2012). However, the challenge, is that CSG water usually requires treatment or amendment prior to its beneficial usage.

CSG water quality varies between coal bed depths, coal formation profiles and basin types (Baker and Slater 2008). Typically, CSG water has a substantial total dissolved solid (TDS) value, with an elevated concentration of Na^+^ ions, but a low concentration of Ca^2+^ and Mg^2+^ (Taulis and Milke 2007). The primary concerns for using CSG co-produced water for irrigation include: high concentration of dissolved salts (that limit plant growth via osmotic drought effects) and an excessive Na^+^ ion concentration (that can cause soil dispersion due to low Ca^2+^ and Mg^2+^ concentration) thereby reducing soil tilth and soil water infiltration rates (Rengasamy and Marchuk 2011). Therefore, treatment or amendment options for CSG waters often consider the reduction of Na^+^ ions.

Some of the more commonly used water treatment technologies used to manage CSG water are (Nghiem et al. 2011):

- desalinisation process using membrane technology;
- distillation;
- electrodialysis and;
- ion exchange resins.

In Australia, the commonly used reverse osmosis (RO) methods generate large volumes of concentrated water (brine) that require additional disposal, resulting in increased capital and operating costs for a CSG well field. Furthermore, the membranes used in the RO system often lack of resistance to fouling (Chapman and Regan 2011), often reducing efficiency and driving up cost; thus making the overall cost for managing CSG waters and gas production unprofitable (Hamawand et al. 2013; Nghiem et al. 2011). Therefore, there is a significant need in the CSG industry for a cost-effective alternative treatment that reduces Na^+^ concentrations on CSG water prior to any beneficial usage.

Natural exchangers such as zeolites are widely used in the treatment of industrial wastewaters for removing contaminants such as Cu^2+^, Fe^3+^, Cr^3+^, Ni^2+^, Cd^2+^, Pb^2+^, NH_4_^+^ amongst other metals (Argun 2008; Bektaş and Kara 2004; Cincotti et al. 2001; Inglezakis et al. 2002; Nguyen and Tanner 1998; Stylianou et al. 2007; Weatherley and Miladinovic 2004). Some authors have also attempted to treat CSG produced waters using natural zeolites (Wang et al. 2012; Zhao et al. 2008, 2009). Natural zeolites are reported to remove sodium ions from solution by replacing them with calcium, thereby reducing Na^+^ concentration. Zhao et al. (2009) suggest that natural exchangers could become a suitable cost effective technology for the treatment of high Na^+^ of CSG co-produced waters.

**Fig. 1:**
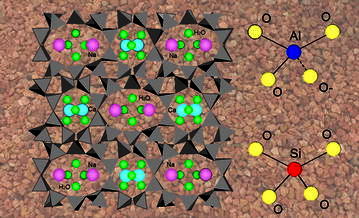
Zeolite three dimensional framework of (SiAl)O_4_ tetrahedral where all oxygen ions of the tetrahedron are shared with adjacent tetrahedral structures (Inglezakis and Zorpas 2012). The presence of Al^3+^ in place of Si^4+^ in the structure gives a negative charge that is balanced by cations. Zeolite material used in this study

Zeolite minerals are natural ion exchangers with a crystalline, porous, three-dimensional aluminosilicate alkali and alkaline metal structures capable of exchanging cations (Pabalan and Bertetti 2001). The zeolite structure is based on a tetrahedral (SiAl)O_4_ framework with four oxygen adjacent shared. The negative surplus charge of the zeolites originates from the substitution of Si^4+^ with Al^3+^, which is then balanced with exchangeable cations such as sodium (Na^+^), potassium (K^+^), calcium (Ca^2+^) or magnesium (Mg^2+^) (Townsend 1986). Natural zeolites have a high cation exchange capacity and selectivity due to their high porosity and sieving properties (Zhao et al. 2008). Clinoptilolite and mordenite zeolite materials have a theoretical cation exchange capacity (CEC) of 202 mEq/100 g (Pabalan and Bertetti 2001) (Fig. 1).

Natural ion exchangers can also be treated from their initial or natural state by single or combined chemical adjustment using acids, bases and inorganic salts (Babak et al. 2013; Günay et al. 2007; Inglezakis et al. 2001; Wang et al. 2012). These chemical treatments result in cation migration from within the crystal framework, and cation replacement by the newly introduced cation species. The chemical treatment removes ions on the exchanger for those that are more removable under ion exchange conditions. Treatment typically increases the presence of one cation on the exchanger converting it into a near homoionic condition (Inglezakis et al. 2004; Semmens and Martin 1988). The homoionic form of the exchanger can improve the effective exchange capacity, enhancing the ion exchange process (Inglezakis et al. 2001; Inglezakis and Zorpas 2012; Vassileva and Voikova 2009; Wang and Peng 2010). Treatment with inorganic salts is recognised as an effective technique to improve natural ion exchangers overall cation exchange capacity (CEC) for water treatment applications.

The present study aims at quantifying the characteristics and ability of Australian natural and treated zeolite for the removal of sodium ions present in co-produced coal seam gas water, as well as the efficacy of zeolite chemical treatment on the adsorption of Na^+^ rate and effective exchange capacity. This work provides an effective and practical application for zeolite on the treatment of CSG waters for Australia and elsewhere.

## Methods

### Material characterisation

The zeolite used in this study had been identified for its suitability in CSG water treatment by another research project being conducted at CQUniversity, Australia (Kele 2015, Unpublished Dissertation). This research project first identified the sodium exchange properties of natural zeolites at a recycled water test site in 2003 (Kele 2005). A variety of volcanic media was tested for sodium exchange properties with CSG water from wells in the Bowen and Surat basins (Kele 2015). The zeolite media in this paper had the best results for sodium reduction in the Bowen basin CSG water (Kele 2015). Samples were crushed and sieved to a size range of 0.6–0.3 mm for subsequent batch type experiments. The natural zeolite was characterised using X-ray fluorescence (XRF) using an ARL SMS-Omega XRF instrument in order to determine its elemental composition, zeolite sample was ground to a pressed pellet for analysis. Characterisation of the zeolite crystalline structure was examined on a XRD PANalytical X’Pert Pro diffractometer (40 kW and 40 mA, angular scanning range 5–80°, and angular speed 2θ/s). Mineral identification was undertaken using X’Pert HighScore search/match software, whilst quantitative analysis of the XRD data was performed using SIROQUANT™ V3. The surface area of the natural zeolite was measured using the N_2_ gas adsorption method at −196 °C on the ASAP2390a from Micrometrics Instrument Corporation, and BET and single point methods were used to determine the specific surface area. Reported mineral characteristics of the material correspond to the mean of two replicates, which did not vary by more than 5 %.

### Water samples

CSG co-produced water samples were collected from a CSG water treatment facility in a gas field in the southern Bowen basin in QLD, Australia, at monthly intervals over a 14 month period. Chemical analyses of the CSG water samples were conducted at Lanfax Laboratories, a laboratory that has National Association of Testing Authorities (NATA) accreditation. Metal concentrations of experimental samples were analysed using an Agilent 720 Inductively Coupled Plasma Optical Emission Spectrometer (ICP-OES), every element was analysed in triplicate for every sample and used by the instrument to estimate relative standard deviation (RSD) automatically (<5 %). ICP multi-element standard solution Merck© was used for calibration purposes. Stock metal standard solutions and chemical reagents used were analytical grade (Chem-Supply). Stock solutions were prepared with Ultrapure Academic Milli-Q water (18.0 Ω). A portable multi-parameter sympHony (VWR) meter with ThermoFisher probes and calibrations solutions were used to determine conductivity (salinity) and pH.

### Batch kinetic experiments and material treatment

The kinetic adsorption of Na^+^ ions was measured for natural and four chemical treated zeolite samples. Natural zeolite was washed with Milli-Q water to remove dust and material impurities, then oven dried at 105 °C for 24 h and stored in sealed HDPE containers. A 40 g sample of natural zeolite was treated with 800 mL of either 1 M calcium chloride (CaCl_2_), 1 M hydrochloric acid (HCl), 1 M potassium chloride (KCl), and 1 M ammonium acetate (NH_4_C_2_H_3_O_2_). In each case, the treatment involved shaking the zeolite samples in contact with the treatment solution for 24 h (incubator Bioline 8500 at 25 °C), rinsing with Milli-Q water (until free of Cl^−^ ions, using silver nitrate (AgNO_3_) test and controlling washing solution salinity), filtration from supernatant (using a mixed cellulose ester membrane filter of 0.45 µm, Advantec^®^), and drying at 105 °C for 24 h.

Batch type kinetic experiments were conducted using 30 g of either natural or treated zeolite material and 600 mL of 0.1 M sodium chloride (NaCl) in a 1000 mL HDPE container placed on an orbital shaker at 300 rpm at a constant temperature of 25 °C for 720 min (incubator Bioline 8500 at 25 °C) (Lehto and Harjula 1995). Aliquots of 1 mL were withdrawn from each container at intervals, where the total sample volume was <2 % of the total volume of the solution (Inglezakis et al. 2004). Aliquots were diluted with HNO_3_ at 2 % for analytical ICP–OES analysis to determine total metal ion concentration. To ensure experimental accuracy, experiments were repeated following the same methods and conditions.

### Adsorption kinetics and diffusion modelling

The concentration of Na^+^ ions transferred to the solid phase of the zeolite, *q* (mEq/100 g), was calculated using Eq. 1:

1 $$ q = (C_{0} - C_{t} ) \times \frac{v}{m} $$

where *C*_0_ and *C*_*t*_ are the amount of initial and retained Na^+^ ions in the solution at time *t* (mEq/L), respectively, *v* is the solution volume (mL) and *m* is the weight of adsorbent (g) (Argun 2008; Kocaoba et al. 2007; Kumar and Jain 2013). Experimental results demonstrate chemical kinetic behaviour describing reaction pathway, time to reach equilibrium and rate of reaction. Determination of kinetic parameters is complex due to the heterogeneity of the adsorption mechanisms within the system (Bektaş and Kara 2004; Oren and Kaya 2006).

The adsorption kinetic models have more than two adjustable parameters that may not be fitted to experimental data by linear regression, requiring a nonlinear least square analysis. For that reason, the sum of error squared (SSE) was used as the minimisation procedure to solve kinetic equations between experimental and predicted data using Matlab^®^ R2012b by MathWorks^®^. Nonetheless, linear fitting was also used and the coefficient of determination (*R*^2^) was calculated using the experimental and predicted data (Bektaş and Kara 2004; Du et al. 2005; Günay et al. 2007). The experimental data was fitted with reaction kinetic models (Table 1) and diffusion models (Table 2) to estimate the adsorption kinetics parameters of the ion exchange system dynamics and diffusion processes under the experimental conditions.

**Table 1:** Reaction kinetic models

Model	Non-linear equation	Linear equation	Model parameters	
Pseudo-first order	$q_{t} = q_{e} (1 - e^{{ - k_{1} t}} )$	$\log (q_{e} - q_{t} ) = \log (q_{e} ) - k_{1} t$	$q_{e} ,K_{1}$	Equation 2
Pseudo-second order	$q_{t} = \frac{{q_{e}^{2} k_{2} t}}{{(1 + q_{e} k_{2} t)}}$	$\frac{t}{{q_{t} }} = \frac{1}{{k_{2} q_{e}^{2} }} + \left( {\frac{1}{{q_{e} }}} \right)t$	$q_{e} ,K_{2}$	Equation 3
Elovich	$q_{t} = \left( {\frac{1}{b}} \right)\ln (abt + 1)$	$q_{t} = \frac{\ln (ab)}{b} + \frac{\ln (t)}{b}$	*a*, *b*	Equation 4

**Table 2:** Diffusion models

Model	Non-linear equation	Model parameters	
Intra-particle diffusion	$q_{t} = k_{i} t^{1/2}+C$	$k_{i}$,$C$	Equation 5
Film diffusion	$D_{f} = 0.23\frac{{r_{0} \delta q_{e} }}{{t_{1/2} }}$	$D_{f}$	Equation 6
Pore diffusion	$D_{p} = 0.03\frac{{r_{0}^{2} }}{{t_{1/2} }}$	*D*_*p*_	Equation 7

The pseudo-first order reaction kinetic model is based on a reversible reaction with an equilibrium state being reached on both liquid and solid phases (Argun 2008; Babak et al. 2013) and it is expressed in Eq. 2. The pseudo-second order kinetic equation is based on the adsorption equilibrium capacity of the solid phase to uptake ions and whose form is expressed in the form of Eq. 3 (Bektaş and Kara 2004; Kumar and Jain 2013). The Elovich equation is widely used in adsorption kinetic studies to describe the chemical adsorption of ions and is written in the form of Eq. 4 (Cincotti et al. 2001; Du et al. 2005).

In Table 1, *q*_*t*_(mEq/100 g) is the amount of Na^+^ ions adsorbed at time *t* (min), *q*_*e*_(mEq/100 g) is equilibrium solid phase concentration, *k*_1_ is first order rate constant for adsorption (min^−1^), *k*_2_ is second order rate constant for adsorption (min^−1^), *a* is the initial adsorption rate (mEq/100 g min) and *b* is the Elovich constant (mEq/100 g).

Kinetics reaction models can describe the adsorption equilibrium, however, they cannot identify the diffusion mechanism of the adsorption processes that is taken place. Therefore, kinetic results can be analysed by using the intraparticle, film and pore diffusion models. Diffusion processes occur when the liquid forms a film layer surrounding the zeolite particle. When the film layer is formed, external diffusion or film diffusion occurs on the surface of the particle. When the liquid reaches the internal framework of the zeolite particle, it is considered an intraparticle diffusion or pore diffusion process (Babak et al. 2013; Karthikeyan et al. 2010).

The examination of ion exchange system kinetics can reveal the adsorption mechanism underlining the sorption processes which can be the product of film diffusion, pore diffusion or both. These two processes provide insight into whether diffusion is controlled by ion exchange or not. Ion exchange kinetics are considered to be a mass transfer process from the liquid phase to the zeolite to determine the time lapsed until equilibrium. When solution with Na^+^ ions is in contact with the natural zeolite, transport of ions occurs from liquid to solid phase through diffusion processes. The rate of adsorption is often limited by the diffusion process on the external surface of the zeolite particle and within the porous sites available in the zeolite (Argun 2008). Equations 5, 6 and 7 (Table 2), determine the intra-particle, film and pore diffusion coefficients of the system.

Model parameters in Table 2, *k*_*i*_ is the intraparticle diffusion rate constant (mEq/100 g min), $C$ is the constant related with the boundary layer (Huang et al. 2010), $D_{f}$ is the film diffusion coefficient (cm^2^/s), $r_{0}$ is the radius of the particle (cm), $\delta$ is the film thickness (cm), $t_{1/2}$ is the half time for the ion exchange process (min) and $D_{p}$ is the pore diffusion coefficient (cm^2^/s) (Argun 2008; Karthikeyan et al. 2010).

### Effective sodium adsorption capacity of natural and treated zeolite material

Effective adsorption capacity is the amount of sodium ions that can be retained in a specific mass of zeolite material and that are exchangeable under specific experimental conditions (Inglezakis 2005). Capacity studies were conducted on batch mode experiment using 30 g of natural and chemical treated zeolite in a 1000 mL HDPE container with 600 mL of NaCl at 0.1 M for 5 days until no further Na^+^ uptake from the zeolite was observed. To ensure experimental accuracy, experiments were repeated using identical conditions.

## Results and discussion

### Material characterisation

The theoretical cation exchange capacity (TEC) of the natural zeolite resulted from the sum of exchangeable cations such as Na^+^, K^+^, Ca^2+^ and Mg^2+^ determined by the chemical composition, in Table 3, was found to be 154 mEq/100 g. Bektaş and Kara (2004) and Inglezakis et al. (2002) found that Turkish and Greek clinoptilolite have a TEC of 250 and 264 mEq/100 g, respectively. The Si:Al ratio for the natural zeolite is 5.29 (mol/mol) and the (Na^+^ + K^+^)/Ca^2+^ ratio is 1.61 (mol/mol). The Si:Al ratio between 4 and 5.5 and are generally characteristic of clinoptilolite zeolite material (Alberti et al. 1997).

**Table 3:** Chemical composition of natural zeolite material used in the present study

Element	${\text{Na}}_{2} {\text{O}}$	${\text{MgO}}$	${\text{Al}}_{2} {\text{O}}_{3}$	${\text{SiO}}_{2}$	${\text{P}}_{2} {\text{O}}_{5}$ ^a^	${\text{SO}}_{2}$ ^a^	${\text{K}}_{2} {\text{O}}$	${\text{CaO}}$	${\text{TiO}}_{2}$	${\text{MnO}}$	${\text{Fe}}_{2} {\text{O}}_{3}$	${\text{BaO}}$	LOI^b^
% (w/w)	1.83	0.88	11.7	72.84	0.04	0.006	1.08	2.85	0.18	0.03	1.34	0.13	7.14

^a^Detection limit (0.001)

^b^LOI determined gravimetrically

The qualitative and quantitative XRD analysis for the mineral crystalline phases of the natural zeolite material determined that it is made clinoptilolite (41 %), mordenite (29 %) and quatrz (30 %). Both clinoptilolite and mordenite made 70 % of the natural zeolite material. The surface area of the natural material measured using the single-point and Brauner–Emmett–Teller (BET) methods with nitrogen gas was 4.47 and 4.50 m^2^/g respectively. Zeolite samples from Vassileva and Voikova (2009) and Bektaş and Kara (2004) were composed of 80 % clinoptilolite and had surface areas of 26 and 15.36 m^2^/g, correspondenly. Furthermore, small amounts of impurities mainly quatz and clays block channels decreasing the estimation of the surface area (Sprynskyy et al. 2010). Therefore, surface are these channels may have a small contribution.

### Characterisation of co-produced coal seam gas water

CSG co-produced water samples were collected from a CSG water treatment facility in the southern part of the Bowen Basin (QLD). The chemical composition of 14 CSG water samples taken at monthly basis are shown in Table 4.

**Table 4:** Chemical analysis of CSG water

	Na^+^	Ca^2+^	Mg^2+^	Cl^−^	${\text{HCO}}_{3}^{ - }$	SAR $\left( {{\text{mEq/mEq}}^{1/2} } \right)$	EC $({\text{dS/cm}})$	pH
$$ \bar{X} $$								
(mg/L)	1156.4	28.3	5.6	1993	618.1	104	6.02	8.34
(mEq/L)	50.3	0.35	0.12	56.2	10.1			
*σ*								
(mg/L)	241.6	38.5	2.8	759.9	140.7	20	1.48	0.58
(mEq/L)	10.5	0.4	0.06	21.4	2.3			

The CSG water chemical composition was dominated by sodium ions, which are 40 times more concentrated when concentrations of Ca^2+^ and Mg^2+^ are compared. The electrical conductivity value greater than 6 dS/cm, pH of 8.34, and the CSG water chemical characterisation, co-produced water from Bowen Basin is classified as brackish alkaline Na^+^–Cl^−^–HCO_3_^−^ water. CSG water chemical composition is consistent with values reported by Hamawand et al. (2013) and Kinnon et al. (2010) for CSG waters from the Bowen Basin, which presented an absence of sulphate, low concentrations of Ca^2+^ and Mg^2+^ and high concentrations of Na^+^, Cl^−^ and HCO_3_^−^ ions.

### Na^+^ adsorption kinetics

In order to determine the adsorption kinetics of Na^+^ onto natural and treated zeolite material, batch type experiments were conducted. Pseudo-first, pseudo-second and Elovich kinetic models were fitted to the experimental data to determine kinetic parameters. Diffusion model and coefficients were determine using intra-particle, film and pore diffusion from experimental data.

### Adsorption kinetics modelling of Na^+^ using zeolite material

**Fig. 2:**
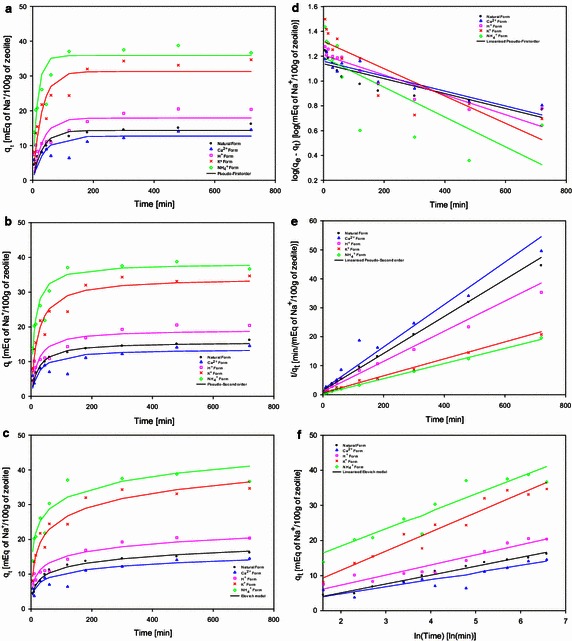
Adsorbed amounts of Na^+^ experimental, modelled and linear form of kinetic data for natural and treated zeolite materials. Initial Na^+^ concentration 0.1 M at pH 7, shaking speed of 300 rpm at 25 °C and solid–liquid ratio 50 g/L. The *symbols* are as follows: (*circle*) Natural zeolite form, (*triangle*) Ca^2+^ form, (*square*) H^+^ form, (*cross*) K^+^ form, (*diamond*) NH_4_^+^ form. **a**, **d** Pseudo-First kinetic order. **b**, **e** Pseudo-Second kinetic order. **c**, **f** Elovich. Each data point is a mean of two replicates, which did not vary by more than 5 %

The removal of Na^+^ ions by ion exchange and adsorption on to zeolite material increased with time and plateau attaining a maximum value as shown by the experimental data in Fig. 2.

Evidently, the adsorption process consisted of two main reaction stages; a fast adsorption followed by a slow adsorption. The fast Na^+^ adsorption process by the natural and treated zeolite material occurred among the 100 min. The rapid process is then followed by a slow adsorption that gradually decreased as contact time increased. After 480 min, the Na^+^ adsorption process almost reached the maximum adsorption capacity under the experimental conditions for natural and treated zeolite material. A similar behaviour was observed and reported by Argun (2008) and Bektaş and Kara (2004) for natural and treated clinoptilolite.

The experimental data was used to determine kinetic constants and predict the kinetic curves of the ion exchange system using pseudo-first, pseudo-second order and Elovich as shown in Fig. 2. Table 5 reports the values obtained from experimental and modelled Na^+^ adsorption capacity of zeolite material, the adsorption rate, capacity, SSE and *R*^2^ found between the experimental and modelled data.

**Table 5:** Experimental and kinetic model coefficients for Na^+^ adsorption on natural and treated zeolite material

Zeolite form	Exp.	Pseudo-first order				Pseudo-second order				Elovich Equation			
	$q_{e}$ (mEq/100 g)	$q_{e}$ (mEq/100 g)	*k*_1_ (min^−1^)	SSE	$R^{2}$	$q_{e}$ (mEq/100 g)	$k_{2}$ (mEq/100 g min)	SSE	$R^{2}$	$a$ (mEq/100 g min)	$b$ (mEq/100 g)	SSE	$R^{2}$
${\text{Natural}}$	16.16	14.34	0.031	4.08	0.96	15.67	0.002	2.27	0.99	2.54	0.39	1.83	0.99
${\text{Ca}}^{2 + }$	15.52	12.71	0.026	6.13	0.87	13.63	0.003	4.91	0.90	2.87	0.49	3.41	0.94
${\text{H}}^{ + }$	20.40	17.90	0.030	10.29	0.84	19.20	0.002	7.72	0.88	27.43	0.20	7.43	0.95
${\text{K}}^{ + }$	34.68	31.29	0.033	12.70	0.91	34.14	0.001	8.76	0.95	6.03	0.18	7.10	0.96
${\text{NH}}_{4}^{ + }$	36.67	35.92	0.063	9.21	0.94	38.28	0.002	4.91	0.98	27.43	0.20	7.43	0.95

The experimental adsorption capacity of natural zeolite form after 720 min was $q_{e} =$ 16.16 mEq/100 g, which is higher that the capacity determined by the kinetic models used (Fig. 2a–c). The pseudo-second model, which is demonstrated to be a model that best describes the Na^+^ adsorption process for natural zeolite under experimental conditions, (Fig. 2e), estimated the capacity after 720 min to be $q_{e} =$ 15.67 mEq/100 g and the adsorption rate of $k_{2} = 0$.002 mEq/100 g min using a NaCl concentration at 0.1 M. The Elovich model showed a high correlation coefficient (Fig. 2f) for natural zeolite form, describing a rapid initial adsorption of $a =$ 2.54 mEq/100 g min and the number of sites available for adsorption of $b =$ 0.39 mEq/100 g min. The results obtained from natural zeolite kinetic modelling (Table 5) were comparable with values report for adsorption of Ni^2+^, Pb^2+^ and ${\text{NH}}_{4}^{ + }$ ions using clinoptilolite in terms of ion exchange adsorption behaviour reported by Argun (2008), Günay et al. (2007), and Nguyen and Tanner (1998).

Natural zeolite was treated with a range of inorganic salts and acid solutions to modify its natural state. These treatments stages resulted in the migration of cations that were naturally contained in the zeolite framework for cations contained in the inorganic and acid treatments. Each treatment introduces only one type of cation that replaces the natural cations contained within the zeolite framework. The treated form of zeolite is known as homoionic (Inglezakis and Zorpas 2012; Wang et al. 2012). Figure 2 shows the experimental adsorption behaviour of Na^+^ ions for different homoionic forms of treated zeolite, as well as the modelled kinetics. The experimental data and kinetic constants obtained are shown in Table 5.

In Fig. 2, the Na^+^ adsorption behaviour for each zeolite treatment is shown. Zeolite material in Ca^2+^ and H^+^ form have an initial adsorption for the first 200 min of contact time with the NaCl solution at 0.1 M. After the rapid initial adsorption, a reduction of the kinetic process is observed from the 300 min until 720 min when adsorption rate is low. In comparison, zeolites treated with KCl and NH_4_C_2_H_3_O_2_ showed a much more rapid adsorption during the first 100 min of contact time followed by a reduction in adsorption kinetic for the following 200 min. In addition, the final Na^+^ adsorption after 720 min is greater for K^+^ and NH_4_^+^ zeolite forms than those observed for Ca^2+^ and H^+^ zeolite forms (Table 5).

Moreover, the adsorption of Na^+^ for K^+^ and NH_4_^+^ zeolite forms after the first 100 min increased by 2.5–3 fold throughout the treatment using KCl and NH_4_C_2_H_3_O_2_ at 1 M. The experimental adsorption capacity of zeolite treated with CaCl_2_ and HCl solutions were found to be *q*_*e*_ = 15.52 mEq/100 g and *q*_*e*_ = 20.40 mEq/100 g, after 720 min respectively. Treatments such as KCl and NH_4_C_2_H_3_O_2_ achieved an adsorption of *q*_*e*_ = 34.68 mEq/100 g and *q*_*e*_ = 36.67 mEq/100 g. The adsorption capacity of Na^+^ ions by NH_4_^+^ zeolite form after 720 min was increased 2.3 fold when compared with Ca^2+^ zeolite form.

The kinetic models fitted the experimental data describing the ion exchange system for each treatment studied (Table 5; Fig. 2). The model that better describes the experimental data for all treatments is the pseudo-second kinetic order and the linearized form of the kinetic is shown in Fig. 2e. The pseudo-second order kinetic model was also the preferred model to describe the ion exchange and adsorption kinetics when clinoptilolite was used to remove lead, nickel and ammonium ions from solutions (Kocaoba et al. 2007; Nguyen and Tanner 1998; Vassileva and Voikova 2009).

The zeolite treatments have shown an improvement on the ion exchange process for adsorption of Na^+^ ions for both adsorption rate and capacity values. The homoionic NH_4_^+^ zeolite form treated with NH_4_C_2_H_3_O_2_ has enhanced the Na^+^ adsorption process when compared with the natural zeolite. The NH_4_^+^ zeolite form has a Na^+^ adsorption capacity of *q*_*e*_ = 36.67 mEq/100 g, while the Na^+^ adsorption capacity of natural zeolite form is *q*_*e*_ = 16.16 mEq/100 g. The experimental Na^+^ adsorption for treated zeolite with 1 M NH_4_C_2_H_3_O_2_ 2.3 folds the value determined for adsorption capacity on natural zeolite. The pseudo-second order kinetic model describes the behaviour obtained experimentally with a coefficient determination of *R*^2^ = 0.98. The linearized form for ${\text{NH}}_{4}^{ + }$ treated and natural zeolite are shown in Fig. 2e. The Na^+^ adsorption rate determined by the pseudo-first order kinetic model and Elovich showed that is the highest among the natural and treated zeolite material with a value of *k*_1_ = 0.063 mEq/100 g min and *a* = 27.47 mEq/100 g min, respectively. Comparable results were reported by Wang et al. (2012), (Argun 2008) and Günay et al. (2007) for clinoptilolite material in which improvement in the adsorption of sodium, nickel and lead ions in solution was possible through the chemical treatment using acids and inorganic salts.

### Diffusion modelling of Na^+^ using zeolite material

In order to determine the diffusion mechanism of the adsorption kinetics of Na^+^ ions by natural and treated zeolite material intra-particle model, film and pore diffusion equation were applied to the experimental data. In the ion exchange process, the adsorption of Na^+^ ions may indeed be controlled by one or more steps, such as film and/or pore diffusion. Usually, film diffusion occurs quickly where ions migrate from the bulk solution to the surface of the zeolite particle creating a liquid film and attaining equilibrium with the available sites on the surface. Film diffusion is followed by the pore diffusion which is a slower process. Intra-particle diffusion was explored in order to determine the intra-particle diffusion rate and the effect of the thickness of the boundary layer on the adsorption of Na^+^ ions for natural and treated zeolite materials (Table 6). The diffusion coefficients for film and pore were calculated to determine which diffusion process limited the kinetics of the ion exchange system for the adsorption of Na^+^ ions (Table 6).

**Table 6:** Intra-particle diffusion model, film and pore diffusion values of Na^+^ ions for natural and treated zeolite material

Zeolite form	Intra-particle diffusion			Film diffusion	Pore diffusion
	*k*_*i*_ (mEq/100 g*min^1/2^)	*C* (mEq/100 g)	*R* ^2^	*D*_*f*_ (cm^2^/s)	*D*_*p*_ (cm^2^/s)
Natural	1.262	1.40	0.99	2.96 × 10^−5^	5.70 × 10^−5^
Ca^2+^	0.43	4.23	0.82	2.66 × 10^−5^	5.70 × 10^−5^
H^+^	0.75	6.14	0.98	3.73 × 10^−5^	5.70 × 10^−5^
K^+^	1.792	7.69	0.93	4.23 × 10^−5^	3.80 × 10^−5^
NH_4_^+^	2.37	10.71	0.94	2.01 × 10^−4^	1.71 × 10^−4^

**Fig. 3:**
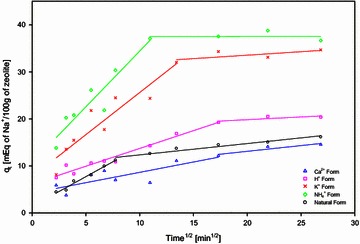
Intra-particle diffusion model of Na^+^ ions and experimental data for natural and treated zeolite materials. Initial Na^+^ concentration 0.1 M at pH 7, shaking speed of 300 rpm at 25 °C and solid–liquid ratio 50 g/L. The *symbols* are as follows: (*circles*) Natural zeolite form, (*triangle*) Ca^2+^ form, (*square*) H^+^ form, (*cross*) K^+^ form, (*diamond*) NH_4_^+^ form

Figure 3 shows the linear representation of the intra-particle diffusion model for natural and treated zeolite. When experimental data is plotted a straight line should be identified in order to assure that the adsorption process of Na^+^ ions is controlled by intra-particle diffusion only. However, experimental data shown in Fig. 3 exhibit multi-linear plots, which indicates that the adsorption process is influenced by two or more steps. From Fig. 3, it is evidenced that the external adsorption is significant only in the early stages of Na^+^ adsorption represented by the first linear sharper portion. The second linear adsorption is the gradual adsorption controlling the intra-particle diffusion.

The first straight portion of the plots in Fig. 3 are assumed to be related with macropore and mesopore diffusion. The second portion represents the micropore diffusion. The slope of the first segment correspond to the intra-particle diffusion constant rate (*k*_*i*_) and the intercept (*C*) of the first segment with the y-axis correspond to the measure of the boundary layer, which are reported in Table 6 for natural and treated zeolite. Nguyen et al. (2015) using Australian iron coated zeolite for adsorption of cadmium, chromium, copper, zinc and lead found that the intra-particle diffusion plot exhibit two linear plots. A fast step attributed to the external diffusion for the first 240 min (15 min^1/2^) followed by slow step endorsed to the intra-particle diffusion till 1500 min (38 min^1/2^) (Nguyen et al. 2015).

Film and pore diffusion coefficients for particles of natural zeolite (0.6–0.3 mm) for experimental conditions were calculated as 2.96 × 10^−5^ and 5.70 × 10^−5^ cm^2^/s, respectively. Often, when film diffusion has a greater value and internal diffusion has lower values the process is believed to be governed by particle diffusion. If pore diffusion coefficient results to be greater than film diffusion, the process is governed by film diffusion (Karthikeyan et al. 2010). Diffusion coefficients calculated for natural zeolite suggested that diffusion process is in some extent rate-limited by the film diffusion indicating the influence of the film diffusion. Film and pore diffusion coefficients were calculated through treatments detailed in Table 6. Film diffusion is not the single rate-limiting factor for treated zeolite. Although, film diffusion coefficients for K^+^ and NH_4_^+^ zeolite forms were smaller than those coefficients found for pore diffusion, which is in accordance with the intra-particle diffusion model.

### Maximum level of sodium adsorption for natural and treated exchangers

**Fig. 4:**
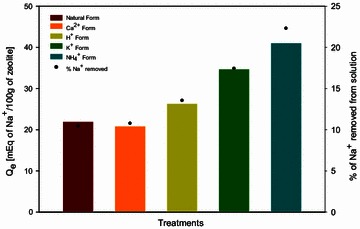
Effect of zeolite treatment on the effective adsorption of Na^+^ ions and the percentage of Na^+^ ions removed. Na^+^ concentration 0.1 M at pH 7, shaking speed of 300 rpm at 25 °C and solid–liquid ratio of 50 g/L. Each data point is a mean of two replicates, which did not vary by more than 5 %

Effective Na^+^ ion adsorption capacity was studied in batch mode until no metal adsorption was observed in the system using natural and treated zeolite materials and NaCl solutions at 0.1 M. Zeolite and the corresponding solution systems had reached equilibrium within the first 5 days of contact time. Figure 4 shows the effective Na^+^ adsorption for natural and treated zeolite as well as the overall percentage of Na^+^ adsorption.

The natural zeolite material exhibited a capacity of *Q*_*e*_ = 22.04 mEq/100 g and 10.4 % of Na^+^ removed from the NaCl solution at 0.1 M (Fig. 4). Zeolites in Ca^2+^ and H^+^ form, showed similar Na^+^ adsorption capacity observed on natural zeolite with values of *Q*_*e*_ = 20.89 mEq/100 g and *Q*_*e*_ = 26.41 mEq/100 g, correspondingly. K^+^ and NH_4_^+^ zeolite forms were found to have higher adsorption capacity values for Na^+^ ions, *Q*_*e*_ = 34.79 mEq/100 g and *Q*_*e*_ = 41.08 mEq/100 g, respectively. Removal of Na^+^ ions by the homoionic K^+^ zeolite form reached 17.4 %, whilst NH_4_^+^ zeolite form achieved a removal of 22.3 %. Maximum adsorption level of Na^+^ ions using NH_4_^+^ zeolite form was 1.8 times higher than the observed for natural zeolite form.

The results in Fig. 4 demonstrate that a zeolite material treated with K^+^ or NH_4_^+^ ions substantially increased the affinity for adsorption of Na^+^ ions. All the results mentioned reveal that the capability of zeolite materials for the adsorption of Na^+^ ions was in accordance with the order NH_4_^+^ > K^+^ > H^+^ > Ca^2+^ zeolite forms. Similar data have also been reported by Petrus and Warchoł (2003) whom confirmed that selectivity of clinoptilolite is weaker for divalent cations and predominantly determined by the hydrated radii of the cations that for NH_4_^+^ ion is 3.3 Å, while Ca^2+^ ions is 4.2 Å.

Table 7 depicts the maximum Na^+^ exchange capacity for the zeolite material for the natural and treated forms. Zeolite treatment using NH_4_C_2_H_3_O_2_ improved the adsorption of Na^+^ ions when compared with the natural zeolite and achieved a 26.6 % of the estimated maximum exchangeable capacity for zeolite material (154 mEq/100 g). These reported values are comparable with other studies in which adsorption of Cu^2+^, Fe^3+^, Cr^3+^, Pb^2+^ ions were tested using zeolites (Inglezakis et al. 2002). In this study, the average maximum level of exchange for zeolite reached 19 % the total exchangeable capacity.

**Table 7:** Maximum Na^+^ exchangeable capacity for natural and treated zeolite

Treatments	Experimental	% of exchangeable capacity
	*Q*_*e*_ (mEq 100 g^−1^)	%
Exchangeable capacity	154	–
Natural	22.04	14.3
Ca^2+^	20.89	13.5
H^+^	26.41	17.1
K^+^	34.79	22.5
NH_4_^+^	41.08	26.6

## Conclusions

The natural ion exchange material is composed of 70 % zeolite type minerals with a blend of 41 % clinoptilolite, 29 % mordenite and 30 % quartz. The theoretical cation exchange capacity determined by the exchangeable cations held on the zeolite material 154 mEq/100 g. The maximum Na^+^ adsorption capacity observed for natural zeolite was 22.04 mEq/100 g, which represents 14.3 % of the exchangeable capacity determined XRF. The capacity of the zeolite material enhanced through treatment using NH_4_C_2_H_3_O_2_ at 1 M concentration, attained a Na^+^ adsorption capacity of 41.08 mEq/g, which represents 26.6 % of the theoretical exchangeable capacity of zeolite material. The adsorption of Na^+^ ions by natural zeolite reached a capacity of 14.34 mEq/100 g after 720 min and an adsorption rate determined by a pseudo-second order kinetic model of *k*_2_ = 0.002 mEq/100 g min. Overall, homoionic treatment of zeolite materials have improved the Na^+^ adsorption rate and capacity. NH_4_^+^ zeolite form presented the largest sodium adsorption capacity and rate after 720 min, which was calculated using a pseudo-second order kinetic model as *q*_*e*_ = 38.28 mEq/100 g and *k*_2_ = 0.002 mEq/100 g min. The higher Na^+^ adsorption by homoionic treated zeolite can be explained by the enrichment of cations on the natural material creating new available sites that may be accessible for incoming cations, hence higher efficiency in total adsorption is observed. Although homoionic treatment enhanced Na^+^ adsorption, the maximum adsorption capacity observed is a portion of the theoretical capacity of the material. This could be explained by the existing impurities in the zeolite material that overestimate the theoretical capacity based on the exchangeable cations.

Intra-particle diffusion model showed that both natural and treated zeolite did not exhibit only intra-particle diffusion mechanism. Furthermore, modelling showed that film and pore diffusion occurred during the Na^+^ adsorption process. Adsorption of Na^+^ ions onto natural and treated zeolite studied has shown that Na^+^ adsorption capability was in accord with the order of zeolites in NH_4_^+^ > K^+^ > H^+^ > Ca^2+^ form.

The treatment of zeolite material shows an increment on the Na^+^ adsorption when it is compared with its natural form. Results indicate that by implementing homoionic treatments higher adsorption rates of Na^+^ ions are achieved. This indicates that Na^+^ ions contained in CSG waters can be removed from the co-produced water reducing the environmental concerns due to high concentrations of sodium ions.
